# Supplementary material for: Multi‐dataset identification of innovative feature genes and molecular mechanisms in keratoconus
Source: J Cell Mol Med. 2024 Sep 19;28(18):e70079. doi: 10.1111/jcmm.70079 (PMC11412914; doi:10.1111/jcmm.70079)
Supplement: Supplementary file 2 — Material S2. [file JCMM-28-e70079-s002.zip › 7.GSEA analysis/1.ARL11/KEGG_ARACHIDONIC_ACID_METABOLISM.html]

Details for gene set KEGG\_ARACHIDONIC\_ACID\_METABOLISM[GSEA]

|  || Dataset | ARL11.group.cls#h\_versus\_l |
| Phenotype | group.cls#h\_versus\_l |
| Upregulated in class | l |
| GeneSet | KEGG\_ARACHIDONIC\_ACID\_METABOLISM |
| Enrichment Score (ES) | -0.27718416 |
| Normalized Enrichment Score (NES) | -0.92891484 |
| Nominal p-value | 0.59591836 |
| FDR q-value | 1.0 |
| FWER p-Value | 1.0 |
Table: GSEA Results Summary

  

Fig 1: Enrichment plot: KEGG\_ARACHIDONIC\_ACID\_METABOLISM      
 Profile of the Running ES Score & Positions of GeneSet Members on the Rank Ordered List

  

| SYMBOL | TITLE | RANK IN GENE LIST | RANK METRIC SCORE | RUNNING ES | CORE ENRICHMENT || 1 | PTGIS | na | 3174 | 0.233 | -0.0519 | No |
| 2 | HPGDS | na | 3609 | 0.215 | -0.0279 | No |
| 3 | CYP2C19 | na | 4519 | 0.182 | -0.0232 | No |
| 4 | PLA2G4E | na | 4751 | 0.174 | -0.0003 | No |
| 5 | GGT6 | na | 5919 | 0.139 | -0.0104 | No |
| 6 | CYP4A11 | na | 6286 | 0.129 | 0.0011 | No |
| 7 | PLA2G3 | na | 6310 | 0.129 | 0.0223 | No |
| 8 | LTC4S | na | 7374 | 0.104 | 0.0094 | No |
| 9 | GPX7 | na | 7609 | 0.100 | 0.0196 | No |
| 10 | PTGDS | na | 7697 | 0.098 | 0.0337 | No |
| 11 | GGT5 | na | 8131 | 0.090 | 0.0365 | No |
| 12 | ALOX15 | na | 8181 | 0.089 | 0.0502 | No |
| 13 | TBXAS1 | na | 8331 | 0.086 | 0.0605 | No |
| 14 | CYP2E1 | na | 8693 | 0.079 | 0.0636 | No |
| 15 | ALOX5 | na | 8741 | 0.079 | 0.0756 | No |
| 16 | ALOX15B | na | 8872 | 0.077 | 0.0850 | No |
| 17 | PLA2G12B | na | 9215 | 0.072 | 0.0873 | No |
| 18 | PTGES | na | 9464 | 0.068 | 0.0916 | No |
| 19 | LTA4H | na | 9911 | 0.061 | 0.0892 | No |
| 20 | GGT7 | na | 11406 | 0.043 | 0.0534 | No |
| 21 | CBR1 | na | 11596 | 0.040 | 0.0548 | No |
| 22 | CYP2B6 | na | 12739 | 0.029 | 0.0268 | No |
| 23 | PLA2G2C | na | 13424 | 0.024 | 0.0111 | No |
| 24 | GPX1 | na | 14044 | 0.018 | -0.0036 | No |
| 25 | EPHX2 | na | 14165 | 0.017 | -0.0041 | No |
| 26 | JMJD7-PLA2G4B | na | 14509 | 0.015 | -0.0115 | No |
| 27 | CBR3 | na | 16718 | -0.001 | -0.0749 | No |
| 28 | PLA2G4A | na | 17245 | -0.005 | -0.0893 | No |
| 29 | CYP2C9 | na | 17344 | -0.006 | -0.0911 | No |
| 30 | PLA2G6 | na | 17692 | -0.008 | -0.0997 | No |
| 31 | PLA2G12A | na | 17822 | -0.009 | -0.1019 | No |
| 32 | CYP2C8 | na | 18873 | -0.016 | -0.1295 | No |
| 33 | PTGES2 | na | 19323 | -0.019 | -0.1391 | No |
| 34 | PLA2G2E | na | 19951 | -0.023 | -0.1532 | No |
| 35 | CYP2C18 | na | 20676 | -0.029 | -0.1692 | No |
| 36 | GPX4 | na | 22138 | -0.041 | -0.2044 | No |
| 37 | GPX3 | na | 22510 | -0.045 | -0.2074 | No |
| 38 | CYP2U1 | na | 22637 | -0.047 | -0.2031 | No |
| 39 | GGT1 | na | 22662 | -0.047 | -0.1958 | No |
| 40 | GPX2 | na | 22726 | -0.047 | -0.1896 | No |
| 41 | CYP2J2 | na | 22727 | -0.047 | -0.1816 | No |
| 42 | PLA2G10 | na | 23788 | -0.060 | -0.2020 | No |
| 43 | GPX5 | na | 24047 | -0.063 | -0.1987 | No |
| 44 | PTGS2 | na | 26519 | -0.099 | -0.2532 | No |
| 45 | ALOX12 | na | 27352 | -0.116 | -0.2575 | Yes |
| 46 | CYP4A22 | na | 27781 | -0.125 | -0.2486 | Yes |
| 47 | PLA2G2D | na | 27985 | -0.130 | -0.2322 | Yes |
| 48 | CYP4F3 | na | 28563 | -0.145 | -0.2242 | Yes |
| 49 | AKR1C3 | na | 30128 | -0.191 | -0.2368 | Yes |
| 50 | GPX6 | na | 30199 | -0.194 | -0.2058 | Yes |
| 51 | CYP4F2 | na | 30243 | -0.196 | -0.1737 | Yes |
| 52 | PLA2G2F | na | 30623 | -0.208 | -0.1493 | Yes |
| 53 | PTGS1 | na | 31481 | -0.239 | -0.1334 | Yes |
| 54 | PLA2G5 | na | 32093 | -0.264 | -0.1061 | Yes |
| 55 | PLA2G2A | na | 32310 | -0.275 | -0.0656 | Yes |
| 56 | PLA2G1B | na | 33526 | -0.362 | -0.0392 | Yes |
| 57 | ALOX12B | na | 34096 | -0.434 | 0.0182 | Yes |
Table: GSEA details [plain text format]

  

Fig 2: KEGG\_ARACHIDONIC\_ACID\_METABOLISM      
 Blue-Pink O' Gram in the Space of the Analyzed GeneSet

  

Fig 3: KEGG\_ARACHIDONIC\_ACID\_METABOLISM: Random ES distribution      
 Gene set null distribution of ES for **KEGG\_ARACHIDONIC\_ACID\_METABOLISM**
